# Association analysis identifies *ZNF750 *regulatory variants in psoriasis

**DOI:** 10.1186/1471-2350-12-167

**Published:** 2011-12-20

**Authors:** Ramon Y Birnbaum, Genki Hayashi, Idan Cohen, Annie Poon, Haoyan Chen, Ernest T Lam, Pui-Yan Kwok, Ohad S Birk, Wilson Liao

**Affiliations:** 1 Department of Bioengineering and Therapeutic Sciences, University of California, San Francisco, California, USA; 2 Institute for Human Genetics, University of California, San Francisco, California, USA; 3 The Morris Kahn Laboratory of Human Genetics, NIBN, Ben-Gurion University, Beer-Sheva, Israel; 4 Department of Dermatology, University of California, San Francisco, California, USA; 5 Cardiovascular Research Institute, University of California, San Francisco, California, USA

## Abstract

**Background:**

Mutations in the *ZNF750 *promoter and coding regions have been previously associated with Mendelian forms of psoriasis and psoriasiform dermatitis. *ZNF750 *encodes a putative zinc finger transcription factor that is highly expressed in keratinocytes and represents a candidate psoriasis gene.

**Methods:**

We examined whether *ZNF750 *variants were associated with psoriasis in a large case-control population. We sequenced the promoter and exon regions of *ZNF750 *in 716 Caucasian psoriasis cases and 397 Caucasian controls.

**Results:**

We identified a total of 47 variants, including 38 rare variants of which 35 were novel. Association testing identified two *ZNF750 *haplotypes associated with psoriasis (p < 0.05). We also identified an excess of rare promoter and 5'untranslated region (UTR) variants in psoriasis cases compared to controls (p = 0.041), whereas there was no significant difference in the number of rare coding and rare 3' UTR variants. Using a promoter functional assay in stimulated human primary keratinocytes, we showed that four *ZNF750 *promoter and 5' UTR variants displayed a 35-55% reduction of *ZNF750 *promoter activity, consistent with the promoter activity reduction seen in a Mendelian psoriasis family with a *ZNF750 *promoter variant. However, the rare promoter and 5' UTR variants identified in this study did not strictly segregate with the psoriasis phenotype within families.

**Conclusions:**

Two haplotypes of *ZNF750 *and rare 5' regulatory variants of *ZNF750 *were found to be associated with psoriasis. These rare 5' regulatory variants, though not causal, might serve as a genetic modifier of psoriasis.

## Background

Psoriasis is a chronic, inflammatory disorder of the skin affecting 2-4% of the Caucasian population [1]. Clinically, psoriasis is characterized by red, scaly plaques typically favoring the elbows, knees, scalp, umbilicus, and gluteal cleft and may be associated with nail dystrophy and arthritis. Histologically, psoriasis is marked by epidermal hyperplasia, dilated vasculature in the dermal papillae, and the presence of T cell infiltrates. Genome-wide association studies and other genetic investigations of psoriasis have identified at least 18 common variants affecting psoriasis susceptibility [2-9]. However, in aggregate, these common variants only explain a fraction of the heritability in psoriasis [10]. Therefore, the missing heritability in psoriasis might be explained by other types of variants not captured by the previous genetic studies, such as rare variants with a low minor allele frequency in the general population.

One candidate susceptibility gene for psoriasis is *ZNF750*, a gene located at chromosome 17q25 within the PSORS2 locus. *ZNF750 *has previously been reported to be associated with autosomal dominant forms of psoriasis or psoriasiform dermatitis in two separate, multigenerational families. The first family was a five-generation Jewish Israeli family of Moroccan descent in which affected members displayed clinical features of both psoriasis and seborrheic dermatitis [11]. The causative mutation was identified as a *ZNF750 *frameshift mutation at residue 19 of this 723-residue protein, resulting in a 44-residue truncated protein which abrogated the zinc finger binding domain. The second family was a five-generation Chinese family with psoriasis [12]. Gene mapping and functional studies identified the most likely causal mutation as a c.-625A>C promoter variant in *ZNF750 *that resulted in 42% reduction in promoter activity.

Since Mendelian-pattern psoriasis in these two large families is associated with *ZNF750*, we sought to examine whether variants in *ZNF750 *might influence the development of psoriasis in a larger population. In this study, we investigated whether variants within the *ZNF750 *promoter, 5' UTR, coding regions, and 3' UTR were associated with psoriasis in a Caucasian population.

## Methods

### Patients

DNA samples from 716 unrelated Caucasian psoriasis cases, 397 Caucasian healthy adult controls, and 20 additional family members were collected from the University of California, San Francisco and Washington University, St. Louis. Cases were recruited from outpatient dermatology clinics and the diagnosis of psoriasis was confirmed by a board-certified dermatologist. Healthy controls, recruited from the local community, reported no history of autoimmune disease or cancer according to a written screening questionnaire. All subjects gave written informed consent for study participation in accordance with the institutional review board at their respective institutions.

### Sequencing

Each DNA sample was sequenced over 6 amplicons to cover the promoter and 3 exons of *ZNF750*. PCR was performed using the following primer pairs, sequences listed 5' to 3': amplicon 1 (531 bp, forward AATACTGTGCCTCCCAGGGTAT, reverse GTACTTACCAGAGGTGGGCAGTG); amplicon 2 (710 bp, forward TGTCCTGACACCAAGACTGC, reverse CGACTGGAACAAATGCAGAA); amplicon 3 (698 bp, forward GGCATCACCCTGCAAGAG, reverse GGTTTAACCTGGAAGGACTCG); amplicon 4 (755 bp, forward CAGGCCAGAGTCTGCATTTT, reverse TGGCTGCCAGGTTTATCTCT); amplicon 5 (673 bp, forward CCCTCAACCTCTCCAAGAAA, reverse GTGGCCGTAGCTCTGTGAAC); amplicon 6 (783 bp, forward CTAACGCCGGGTTCACAC, reverse GCACCCGTTCACAGGTTAAT). PCR was performed using 8 ng genomic DNA, 0.4 μM each forward and reverse primer, 1x buffer, 4 mM dNTPs, and 0.3U Qiagen (Valencia, CA USA) HotStar Taq and 1x Q Solution in a 10 μL reaction. PCR was cleaned up by incubation with 1x SAP PCR Clean-Up Reagent (PerkinElmer Life Sciences, Inc. Waltham, MA USA) at 37°C for one hour. Sequencing reactions contained 2.5 μL of clean PCR product, 0.375 μM primer and 8.3% Applied Biosystems (ABI; Foster City, CA USA) BigDye Terminator v3.1 in a 12 μL reaction. Excess dye terminator removal was performed with genCLEAN (Genetix; New Milton, Hampshire, United Kingdom) plates following manufacturer's instructions before sequencing on an ABI 3730xL DNA Analyzer. Sequencing was performed in one direction except for amplicon 6 in which the presence of multiple indels required the use of bidirectional sequencing, using the internal reverse primer AGCCTCTTGATGTTTGTGTGTT and internal forward primer TGGTTGTAAAAACACCTGAATGA. All rare promoter and 5' UTR variants were sequenced a second time in the forward and reverse directions to confirm their accuracy.

### Statistical Methods

Sequencing traces were analyzed with Sequencher (Gene Codes; Ann Arbor, MI USA). Hardy-Weinberg equilibrium p-values were calculated in Haploview to assess sequencing quality and a p-value of 0.05 was used as the significance threshold for exclusion. Individual polymorphisms were tested for association with psoriasis using Fisher's exact test implemented in PLINK. Haploview was used to identify haplotypes using the confidence intervals method and to calculate haplotype associations using a chi-square test. Haploview was also used to perform empirical p-value estimation, using 100,000 permuted experiments. False discovery rate (FDR) q-values were calculated in R. A weighted sum statistic was used to test functionally similar groups of rare variants for association with psoriasis. This approach of combining multiple independent signals has been shown to be significantly more powerful than variant-by-variant or other approaches for the analysis of rare variants [13]. 10,000 permutations were performed to determine significance for each group tested.

### 5' Rapid Amplification of cDNA Ends (5' RACE)

SMART RACE cDNA amplification kit (BD Biosciences; Franklin Lakes, NJ USA) was used to perform 5'-RACE of *ZNF750 *gene. RACE-ready cDNA was synthesized according to the manufacturer's instructions. RACE PCR was performed with gene-specific primers that were used for RT-PCR of *ZNF750 *(Exon 2, GGAACTCGATCCTGCTCTGA). RACE products were excised from the agarose gel, purified, and cloned into pGEM-T easy vector (Promega Corp; Madison, WI USA). Cloned inserts were sequenced with the T7 and SP6 primers and the DNA sequences obtained were compared to published sequences.

### Reporter Constructs for ZNF750 Promoter Assay

The *ZNF750 *reference sequence was taken from National Center for Biotechnology Information, with accession number NM_024702. Five candidate mutations in the promoter and 5' UTR region of *ZNF750 *were analyzed for changes in luciferase reporter activity. Four novel variants were identified in patients diagnosed with psoriasis and one mutation, c.-625A>C was identified by Yang et al [12]. An 849 bp insert containing the promoter and 5' UTR (exon 1 portion) of *ZNF750 *was amplified from human genomic DNA using the primers 5'-CGGCTAGCCAGCAAGCAAGCAGTTTTGGT-3' (*NheI*) and 5'-CCAAGCTTGGATGTGGCCGGTCTTGGT-3' (*HindIII*), and cloned into pGL3 Basic vector (Promega) using *NheI *and *HindIII *restriction enzymes. QuikChange II XL Site-Directed Mutagenesis Kit (Agilent Technologies) was used to create the 5 variations of interest individually. All vectors were sequenced to verify the location of the variation.

### Transfection and Luciferase Assay

Primary keratinocytes were provided by Dr. Dennis Oh and Dr. Susana Ortiz-Urda (University of California San Francisco, San Francisco, CA). The cells were grown in 500 ml Medium 154CF and Human Keratinocyte Growth Supplement (Invitrogen; Carlsbad, CA USA), and 35 μM of CaCl_2_. A total of 10^5 ^cells were seeded in each well of a 6-well plate and were transfected after 24 hours with 500 ng of each reporter construct along with 10 ng of pRL-TK vector (Promega) containing the Renilla luciferase gene as an indicator for normalization of transfection efficiency. Transfections were performed using Lipofectamine™ LTX with PLUS™ Reagent (Invitrogen), according to the manufacturer's instructions. After 24 hours, the cells were stimulated with 250 ng phorbol-12-myristate-13-acetate (PMA) for the PMA positive set of experiments. Cells were incubated for an additional 24 hours and then analyzed for luciferase activity with the Dual-Luciferase^® ^Reporter Assay System (Promega) and Synergy 2 (BioTek; Winooski, VT USA). Firefly luminescence was normalized to Renilla luminescence and reported as relative luciferase units compared to wild-type sequence. All experiments were performed independently three times using primary keratinocytes from a single donor.

## Results

### Identification of ZNF750 Variants in Cases and Controls

To determine whether *ZNF750 *is associated with psoriasis, we sequenced *ZNF750 *in 716 Caucasian psoriasis cases and 397 Caucasian controls. We sequenced the *ZNF750 *promoter region (defined as 400 bp upstream of the transcription start site), 5' UTR, coding regions which include exons 2 and 3, and 3' UTR. In total, we identified 9 common variants (minor allele frequency (MAF) ≥ 2% in the controls, Table 1) and 38 rare variants (MAF < 2%, Table 2). One polymorphism, a novel 8 bp deletion at position 78381172 (hg18) in the 3' UTR, was out of Hardy-Weinberg equilibrium and was not included. All identified polymorphisms were successfully genotyped in greater than 94% of samples and the mean genotyping rate per variant was 97.3%. Nearly all variants were single nucleotide variants (SNVs) with the exception of two single base deletions, one located in the 3' UTR (rs71918228) and the other in the 3' downstream region (rs35156590). All of the identified common variants were present in dbSNP Build 133 whereas 35 of 38 (92%) of the rare variants were novel.

**Table 1:** Common variants identified in *ZNF750*

Name	Position	Property (PolyPhen2 impact)	Alleles	F_case	F_control	Fisher P
rs3744165	78383731	Exon 2, 5' UTR	C/A	0.171	0.161	0.541

rs12450046	78383677	Exon 2, 5' UTR	C/T	0.186	0.172	0.478

rs8074277	78382917	Exon 2, M235V (benign)	A/G	0.189	0.18	0.640

rs35653278	78382757	Exon 2, P288L (probably)	C/T	0.092	0.09	0.937

rs34188981	78382558	Exon 2, T354T	C/T	0.021	0.021	1.000

rs12948179	78381781	Exon 3, P566P	T/C	0.393	0.42	0.233

rs12938126	78381754	Exon 3, A575A	A/G	0.392	0.42	0.232

rs71918228	78381176-79	Exon 3, 3' UTR	CAAA/-	0.465	0.478	0.586

rs35156590	78380584	3' Downstream	-/T	0.369	0.418	**0.028**

Common variants are defined as those with minor allele frequency (MAF) ≥ 2% in controls. Position is on Chr17 (hg18). UTR, untranslated region. PolyPhen2 impact given as benign, possibly damaging, or probably damaging. Alleles are given as major/minor on the (-) strand. F_case, MAF in cases; F_control, MAF in controls.

**Table 2:** Rare variants identified in *ZNF750*

Name	Position	Property (PolyPhen2 impact)	Alleles	F_case	F_control	Fisher P
Novel_1 (c.-597C>T)	78391506	Promoter	C/T	0 of 1312	1 of 745	0.363

Novel_2 (c.-458G>A)	78391367	Promoter	G/A	1 of 1309	0 of 746	1.000

Novel_3 (c.-261G>A)	78391170	Exon 1, 5' UTR	G/A	1 of 1309	0 of 746	1.000

Novel_4 (c.-233 C>T)	78391142	Exon 1, 5' UTR	C/T	4 of 1306	0 of 746	0.303

Novel_5 (c.-232G>A)	78391141	Exon 1, 5' UTR	G/A	1 of 1311	0 of 746	1.000

Novel_6 (c.-45G>C)	78383664	Exon 2, 5' UTR	G/C	1 of 1349	0 of 746	1.000

Novel_7 (c.-36A>G)	78383655	Exon 2, 5' UTR	A/G	1 of 1349	0 of 746	1.000

Novel_8 (c.-32G>A)	78383651	Exon 2, 5' UTR	G/A	1 of 1349	0 of 746	1.000

Novel_9	78383521	Exon 2, T33T	T/C	1 of 1349	0 of 746	1.000

Novel_10	78383438	Exon 2, R61Q (possibly)	G/A	2 of 1348	1 of 745	1.000

Novel_11	78383402	Exon 2, P73L (probably)	C/T	0 of 1350	1 of 745	0.357

Novel_12	78383393	Exon 2, T76I (benign)	C/T	0 of 1350	1 of 745	0.357

Novel_13	78383325	Exon 2, D99N (probably)	G/A	1 of 1349	1 of 745	1.000

Novel_14	78383272	Exon 2, E116E	G/A	1 of 1349	0 of 746	1.000

Novel_15	78383216	Exon 2, A135E (benign)	C/A	0 of 1350	1 of 745	0.357

Novel_16	78383196	Exon 2, A142T (possibly)	G/A	1 of 1349	0 of 746	1.000

Novel_17	78382899	Exon 2, E241Q (probably)	G/C	1 of 1357	0 of 752	1.000

Novel_18	78382882	Exon 2, F246F	T/C	0 of 1358	1 of 751	0.356

rs35283702	78382791	Exon 2, G277R (probably)	G/A	15 of 1343	7 of 745	0.825

Novel_19	78382759	Exon 2, H287Q (possibly)	C/G	1 of 1357	0 of 752	1.000

Novel_20	78382489	Exon 2, F377L (benign)	C/G	0 of 1374	1 of 763	0.359

rs34687659	78382445	Exon 2, Q392R (possibly)	A/G	0 of 1374	1 of 763	0.359

Novel_21	78382401	Exon 2, A407T (probably)	G/A	1 of 1373	0 of 764	1.000

Novel_22	78382375	Exon 2, P415P	G/A	2 of 1372	2 of 762	0.622

Novel_23	78382326	Exon 2, D432H (possibly)	G/C	2 of 1372	0 of 764	0.540

Novel_24	78382188	Exon 2, V478I (benign)	G/A	0 of 1374	1 of 763	0.359

Novel_25	78382160	Intron 2	C/A	1 of 1373	0 of 764	1.000

Novel_26	78382155	Intron 2	A/C	1 of 1373	0 of 764	1.000

Novel_27	78382047	Intron 2, 5 bp from exon 3	T/C	1 of 1373	0 of 764	1.000

rs35792712	78382015	Exon 3, P488P	T/G	0 of 1374	1 of 763	0.359

Novel_28	78381714	Exon 3, G589R (benign)	G/C	1 of 1365	0 of 766	1.000

Novel_29	78381508	Exon 3, A657A	G/A	1 of 1365	0 of 766	1.000

Novel_30	78381034	Exon 3, 3' UTR	G/A	1 of 1357	0 of 756	1.000

Novel_31	78380851	Exon 3, 3' UTR	A/G	1 of 1365	2 of 752	0.290

Novel_32	78380743	Exon 3, 3' UTR	A/G	2 of 1364	0 of 754	0.541

Novel_33	78380697	Exon 3, 3' UTR	T/G	1 of 1365	0 of 754	1.000

Novel_34	78380590	3' Downstream	T/C	2 of 1318	1 of 751	1.000

Novel_35	78380548	3' Downstream	G/A	17 of 1305	9 of 743	1.000

Rare variants are defined as those with minor allele frequency (MAF) < 2% in controls. SNPs Novel_1 through Novel_8 are additionally named by the nucleotide position relative to the translation start site with respect to the cDNA reference. Position is on Chr17 (hg18). UTR, untranslated region. PolyPhen2 impact given as benign, possibly damaging, or probably damaging. Alleles are given as major/minor on the (-) strand. F_case, MAF in cases; F_control, MAF in controls.

### Association Testing of Identified Variants

To analyze the significance of the identified *ZNF750 *variations/mutations in psoriasis patients, we first tested each variant alone using a Fisher's exact test. For the common variants (Table 1), only rs35156590 in the immediate 3' downstream region showed a significant association with psoriasis (p = 0.028, OR 0.81 [95% CI 0.68-0.98]). However, adjustment for multiple hypothesis testing using empirical permutation testing of this SNP yielded p = 0.17. When tested individually, none of the rare variants (Table 2) achieved a significance association with psoriasis (all p > 0.05).

Next, we evaluated whether common variant haplotypes of *ZNF750 *were associated with psoriasis. Two 8-marker haplotypes were significantly associated with psoriasis (Table 3), the risk haplotype CCACCCG(-) (frequency = 0.019, p = 0.0011, OR 3.70 [1.45-9.46]) which includes the risk allele of rs35156590, and the protective haplotype CCACCCGT (frequency = 0.374, p = 0.0106, 0.79 [0.66-0.94]) which contains the non-risk allele of rs35156590. Both of these haplotypes remained significant after correction for multiple comparisons using empirical permutation testing (p = 0.0024 and p = 0.0311, respectively) as well as by calculation of the false discovery rate (q = 0.0077 and q = 0.037, respectively).

**Table 3:** Haplotype association testing between cases and controls

Haplotype	Hap Freq	F_case	F_control	P Value	Emp P-Val	Odds Ratio [95% CI]
CCACCCGT	0.374	0.354	0.41	**0.0106**	**0.0311**	**0.79 [0.66-0.94]**

CCACCTA(-)	0.246	0.253	0.234	0.3321	0.9359	-

ACACCTA(-)	0.137	0.138	0.136	0.9158	1.0000	-

CTGCCTA(-)	0.089	0.091	0.085	0.6426	0.9995	-

CTGTCTA(-)	0.089	0.091	0.086	0.7198	0.9997	-

ACACTTA(-)	0.02	0.02	0.02	0.9666	1.0000	-

CCACCCG(-)	0.019	0.026	0.006	**0.0011**	**0.0024**	**3.70 [1.45-9.46]**

The eight marker haplotype, as ordered in the table, consists of: rs3744165, rs12450046, rs8074277, rs35653278, rs34188981, rs12948179, rs12938126, rs35156590. Hap Freq, overall haplotype frequency; F_case, haplotype frequency in cases; F_control, haplotype frequency in controls; Emp P-Val, empiric p-value calculated by permutation testing.

Due to the well-recognized difficulty in assessing the significance of rare variants on a variant-by-variant basis due to power limitations [14], we utilized a weighted sum statistic [13] to evaluate functional groups of rare variants for psoriasis association. We tested the following rare variant groups: 5' regulatory variants (promoter and 5' UTR), all coding variants, non-synonymous variants, predicted deleterious non-synonymous variants, 3' UTR variants, and all rare variants. We found that only the 5' regulatory variants trended towards a significant association with psoriasis (10 variants in cases, 1 variant in controls, unadjusted p = 0.041, Bonferroni threshold p = 0.008, Table 4). The putative association of the rare 5' regulatory variants with psoriasis was not secondary to their occurrence on a risk haplotype background. Of the 10 patients with rare 5' regulatory variants, only 1 patient was heterozygous for the risk haplotype CCACCCG(-), while 7 patients were actually heterozygous for the protective haplotype CCACCCGT.

**Table 4:** Groupwise association testing of *ZNF750 *functional groups with psoriasis

Rare Variant Group	Total Variants, Cases (n = 716)	Total Variants, Controls (n = 397)	P-value, Weighted Sum Statistic
5' Regulatory (Promoter + 5' UTR)	10	1	**0.041**

All Coding	30	19	0.575
- Non-synonymous	25	15	0.560
- Predicted deleterious	24	11	0.314

3' UTR	5	2	0.351

All Rare	67	32	0.192

### Alternative splicing of ZNF750 mRNA

**Figure 1:**
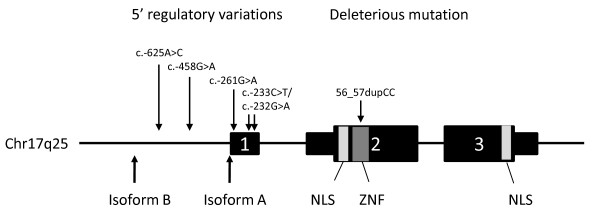
***ZNF750 *gene structure and variants**. *ZNF750 *consists of 3 exons, with the translation start site within exon 2. Narrow boxes indicate 5' and 3' untranslated regions; wider boxes indicate translated regions. *ZNF750 *contains two nuclear localization sites (NLS) and a C2H2 zinc finger domain (ZNF). Arrows depict the locations of a deleterious frameshift mutation seen in a Jewish Moroccan family (56_57dupCC), promoter and 5' UTR variations examined in this study (c.-625A>C, c.-458G>A, c.-261G>A, c.-233 C>T, c.-232G>A), and start sites of *ZNF750 *mRNA isoforms A and B.

In order to characterize the *ZNF750 *promoter and transcripts, the size of *ZNF750 *mRNA was verified using two methods: reverse transcriptase PCR (RT-PCR) and 5' Rapid Amplification of cDNA Ends (5' RACE). Using RT-PCR, the predicted cDNA segments were amplified from mRNA of normal individual skin biopsies. Comparison of the cDNA sequences to NCBI and UCSC databases indicated that there are no alternative splicing variants. 5' Rapid Amplification of cDNA Ends performed for *ZNF750 *in primary keratinocytes produced four 5' RACE PCR products that were cloned and sequenced (data not shown). Alignments of the sequences of the 5' RACE PCR products with human genomic DNA and known mRNA sequences indicated that only two of the four products were specific to *ZNF750*. The short product represents the mRNA isoform A as in Refseq gi13375990; NM_024702.1. The long product, previously demonstrated in cDNA from tongue tumor tissue [GenBank:DA436414][15] represents the mRNA isoform B and includes an additional 500 bp of sequence upstream to exon 1 of mRNA isoform A (Figure 1). The expression levels of the 5'- RACE isoforms were studied by RT-PCR with forward primers specific to isoform A and isoform B, respectively, and a reverse primer specific to exon 2. RT-PCR analyses of several cell lines (including primary keratinocytes) showed that the most abundant variant of *ZNF750 *transcript corresponds to isoform A. Analysis of all 3 reading frames indicated that isoform A and B encode an identical protein: the addition of 500 bp upstream in isoform B compared to isoform A does not result in a different protein. Further investigation of the protein size using Western Blot demonstrated that *ZNF750 *codes for a single protein of 100 KDa (data not shown). Our data indicate that mRNA isoform A of *ZNF750 *is the predominant form of *ZNF750 *expressed in keratinocytes and that the 5' region DNA variants identified in this study lie within the promoter region and 5' UTR of isoform A.

### Functional evaluation of ZNF750 5' Regulatory Variants

**Figure 2:**
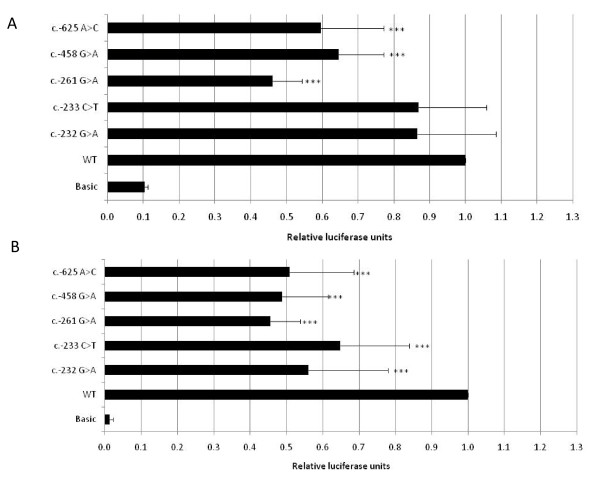
**Luciferase activity of *ZNF750 *regulatory region in human primary keratinocytes**. Bar chart representing luciferase activity of pGL3 constructs containing ZNF750 promoter and 5' UTR variants relative to wild type promoter (WT). Four variants (c.-458G>A, c.-261G>A, c.-233C>T, c.-232G>A) were detected in psoriasis patients in this study, one variant (c.-625A>C) was detected in a psoriasis family in previous study, and a pGL3 basic (empty vector) was used as a negative control. Constructs were co-transfected with Renilla luciferase into primary human keratinocytes and then treated for 24 h with culture medium (A) or culture medium supplemented with 250 ng/ml PMA (B). After 48 h, cells were assayed for luciferase activity. A reduction in luciferase activity is seen for all 4 promoter and 5' UTR variants as well as the c.-625A>C external control. The results represent the means ± SD of three independent experiments. *** p < 0.001 compared to WT by t-test.

To assess whether the *ZNF750 5' *regulatory variants affect *ZNF750 *expression levels, we conducted a promoter function assay in human primary keratinocytes. Seven rare variants in the 5' regulatory region of *ZNF750 *were seen in the psoriasis cases and none of these were present in the controls (Table 2). All of these variants were singletons with the exception of c.-233C>T, which was present in 4 unrelated psoriasis cases. None of these were present in individuals of European descent in the 1000 genomes database. We constructed *ZNF750 *5' regulatory region fragments with 4 of the variants cloned into individual luciferase reporter assay constructs. The 4 selected variants are located in either the *ZNF750 *promoter region (c.-458G>A) or *ZNF750 *untranslated first exon (c.-261G>A, c.-233C>T, c.-232G>A) (Figure 1). Each construct was transfected into human primary keratinocytes which are known to highly express *ZNF750 *[11]. As an external control, we also tested the activity of the c.-625A>C variant which was shown to reduce promoter activity in the Taiwanese familial psoriasis study [12]. We found that the c.-458G>A promoter variant and the c.-261G>A 5' UTR variant showed a significant reduction of 35% and 54% in promoter activity, respectively (p < 0.001). Both c.-233C>T and c.-232G>A in the 5' UTR showed a non-significant reduction of 13% in promoter activity (Figure 2A). Our external control, the c.-625A>C variant, showed a similar significant reduction of 40% in promoter activity as previously published [12]. Interestingly, human primary keratinocytes that have been stimulated with phorbol 12-myristate 13-acetate (PMA) have about 10 fold higher *ZNF750 *promoter activity than unstimulated keratinocytes. Therefore, we tested the effects of the 4 variants on promoter activity after stimulation of human primary keratinocytes with PMA. We found that all 4 of the variants, as well as the external control, showed a significant reduction in promoter activity of 35-55% compared to the wild type promoter (all p < 0.001, Figure 2B).

### *Clinical Phenotype of Psoriasis Patients with 5' Regulatory Variants*

To assess whether psoriasis patients with 5' regulatory variants have a similar clinical phenotype, we clinically characterized the psoriasis patients with the *ZNF750 *5' variants (Table 5). Of the 7 affected patients with detailed clinical information available, 5 reported involvement of the scalp or inverse skin folds, and a negative history of psoriatic arthritis, similar to the sebo-psoriasis, arthritis-negative phenotype previously described in the Israeli-Moroccan family with *ZNF750 *mutation [11].

**Table 5:** Clinical characteristics of subjects with rare *ZNF75*0 variants in the 5' regulatory region

*ZNF750 *Variant	# Pso Cases	Age: Study Entry (Onset)	Gender	PsA	Family History of Psoriasis	Description
c.-458G>A	1	NA	Female	Yes	Sister	Confirmed psoriasis, no other information available

c.-261G>A	1	57 (35)	Male	No	Mother, brother, daughter, niece, nephew	Confirmed psoriasis

c.-233C>T	4	41 (25)	Female	No	Mother, sister	Plaque and inverse psoriasis affecting scalp, trunk, extremities, breast folds, axillae, and genitals.
		32 (15)	Female	No	Mother	Plaque psoriasis with 10-20% BSA affecting scalp, trunk, arms, legs
		34 (5)	Male	No	Father, sister	Guttate psoriasis affecting the scalp, trunk, and arms with 5-10% BSA
		54 (35)	Female	No	Sister	Plaque psoriasis affecting elbows, knees, and legs with 10-20% BSA

c.-232G>A	1	43 (30)	Male	No	Maternal cousin	Moderate plaque and inverse psoriasis affecting scalp, elbows, legs, axillae, genitals, and nails

c.-45G>C	1	30 (5)	Female	No	Father	Moderate guttate (originally plaque) and inverse psoriasis affecting scalp, arms, legs, trunk, axillae, and genitals

c.-36A>G	1	40 (25)	Female	No	Sister	Moderate psoriasis affecting trunk, legs

c.-32G>A	1	65 (NA)	Male	No	NA	Confirmed psoriasis

All psoriasis cases were of Caucasian ethnicity. PsA, psoriatic arthritis diagnosed by rheumatologist; BSA, affected body surface area. NA, information not available.

## Discussion

To our knowledge, this is the first large resequencing study of the *ZNF750 *gene in psoriasis. As *ZNF750 *resides in the PSORS2 region [16-19] and has been previously reported to cause familial psoriasis forms of psoriasis [11,12], we were motivated to evaluate *ZNF750 *in a large cohort of cases and controls. We sequenced a total of 1,113 individuals (716 Caucasian psoriasis cases and 397 ethnically matched healthy controls) in the promoter and exonic regions of *ZNF750 *and identified 47 polymorphisms, of which 35 were novel. According to estimates of Ionita-Laza et al [20], our study captured greater than 99% of the variants with frequency greater than 0.001 in the CEPH population. However, for perspective, we would have needed to sequence 3,521 individuals to capture 100% of the variants with frequency greater than 0.001.

We conducted an association analysis of our identified variants and found no evidence that individual common polymorphisms (MAF > 2%) were associated with disease. Similarly, imputation of HapMap3/1000 Genomes SNPs using psoriasis GWAS data [7] in the interval 200 kb upstream to 200 kb downstream of *ZNF750 *did not find any SNPs with association p-values less than 1.0 × 10^-3 ^(data not shown), suggesting that no common *ZNF750 *SNPs are causal for psoriasis. However, our finding that two *ZNF750 *haplotypes were significantly associated with psoriasis suggests the possibility that other SNPs on 17q in LD with these haplotypes could possibly be associated with psoriasis. We also detected several rare, non-synonymous, potentially deleterious (PolyPhen2) coding variants in *ZNF750 *that were only present in cases and not in controls, including A142T, E241Q, H287Q, and A407T (Table 2). However, given that each of these variants was only detected in a single case and absent additional functional data, it is difficult determine whether these variants were truly deleterious. Therefore, in order to more rigorously evaluate the possible association of rare variants in *ZNF750 *with psoriasis, we used the weighted-sum approach of Madsen and Browning [13] to evaluate functional groups of rare variants for disease association. This method has been found to be robust compared to other rare variant analysis approaches such as CAST, CMC, Variable Threshold, and Li-Leal [13,21]. We found that variants in the promoter or 5' UTR of *ZNF750 *were enriched in the cases compared to controls (10 variants vs 1 variant, p = 0.041). Interestingly, the 1 variant found in a control but in none of the cases was c.-597C>T, which was previously reported to be present in 1 of 85 sporadic psoriasis patients screened and 0 of 188 normal controls [12]. In the same study, the promoter variant c.-625A>C was linked to a multigenerational Taiwanese psoriasis family and was reported to reduce *ZNF750 *promoter activity by 42% [12]. We therefore investigated the functional impact of 4 of our identified 5' regulatory variants in a promoter activity assay. We found that all 4 variants, as well as the c.-625A>C external control, displayed decreased promoter activity compared to the wild-type promoter. The observed effect on *ZNF750 *promoter activity was more pronounced in the presence of the stimulator PMA, suggesting that the biological effects of these variants could be dependent on environmental variables such as immune stimulation or response to external cues.

We obtained DNA from the family members of 6 probands with the rare 5' regulatory *ZNF750 *variants. In a segregation analysis examining the transmission pattern of the rare variants in these six families, we did not identify a clear autosomal dominant pattern (data not shown). Considered together with our functional data, two interpretations could be considered. The first is that certain 5' regulatory variants in *ZNF750 *could serve as a genetic modifier of the psoriasis phenotype or act as an incremental risk modifier similar to the common susceptibility alleles previously identified [4-8]. It should be noted that the HLA-Cw6 allele, which confers the strongest known risk on psoriasis susceptibility, has a penetrance of only 10% [22]. The existence of rare variants that influence psoriasis risk but which have moderate, non-Mendelian effect sizes is certainly possible but difficult to prove. Alternatively, it is possible that there is no true association of 5' regulatory variants in *ZNF750 *with psoriasis and that the nominal association observed is the result of type I error. In this case, the functional effects of these variants on *ZNF750 *promoter activity demonstrated here may not translate into biological relevance for psoriasis. However, of the 7 patients identified in this study with *ZNF750 *5' regulatory variants and for whom detailed clinical descriptions were available, 5 reported scalp psoriasis and 3 reported inverse psoriasis (axillae, genitals), which is reminiscent of the sebo-psoriasis phenotype in the previously reported Morrocan family with a frame shift *ZNF750 *mutation [11]. Variants in the promoter and 5' UTR of *ZNF750 *which decrease *ZNF750 *expression might therefore be associated with a seborrheic form of psoriasis.

*ZNF750 *encodes a 723 amino acid protein that contains two nuclear localization sites (NLS), and two histidines and two cysteines that might serve as zinc binding domains. ZNF750 is a putative member of the C2H2 subclass of zinc finger transcription factors. *ZNF750 *is highly expressed in keratinocytes, which are the major skin cell type affected both in seborrheic dermatitis and in psoriasis. In addition, human primary keratinocytes that have been differentiated with Ca^2+ ^have increased *ZNF750 *promoter activity at levels similar to PMA stimulated cells (unpublished data), suggesting *ZNF750 *may serve an important function in keratinocyte differentiation or immune response in the skin. As keratinocytes in psoriasis or seborrheic dermatitis secrete factors that recruit cells of the immune system and help maintain the inflammatory response [23,24], it is possible that an insufficient level of *ZNF750 *could lead to a downstream effect that fails to repress a stimulated immune response in psoriasis or seborrheic dermatitis.

## Conclusions

In summary, we have performed the first re-sequencing study of the candidate psoriasis gene *ZNF750 *in a large case-control population. Although no individual variants were found to associate with psoriasis, two *ZNF750 *haplotypes showed a significant association. We also observed a nominal association between rare variants in the 5' regulatory region of *ZNF750 *and psoriasis. Collectively, these rare 5' regulatory variants were seen in 10 of 716 (1.4%) of the psoriasis population surveyed. Functional assays demonstrated that 4 of these variants decreased *ZNF750 *promoter activity in accordance with previous reports in psoriasis [12] and psoriasiform dermatitis [11]. We found that these variants did not segregate with the psoriasis phenotype within families, suggesting that they are modifier variants rather than causal for psoriasis. Further studies are warranted to determine whether these rare, non-coding variants could play an influencing role in disease expression.

## Competing interests

The authors declare that they have no competing interests.

## Authors' contributions

RYB conceived the study, performed and supervised the functional work, and helped write the manuscript. GH performed the luciferase assays and helped write the manuscript. IC performed functional characterization of the promoter region. AP performed the sequencing. HC performed statistical analysis. EL performed statistical analysis. PK provided logistical and financial support. OSB supervised some of the functional work and provided financial support. WL designed the study, contributed cases and controls, supervised the sequencing and analysis, helped write the manuscript, and provided financial support. All authors have read and approved the final manuscript.

## Pre-publication history

The pre-publication history for this paper can be accessed here:

http://www.biomedcentral.com/1471-2350/12/167/prepub
